# Fiber-Optic Fluoroimmunoassay System with a Flow-Through Cell for Rapid On-Site Determination of *Escherichia coli* O157:H7 by Monitoring Fluorescence Dynamics

**DOI:** 10.3390/bios3010120

**Published:** 2013-03-08

**Authors:** Kumiko Miyajima, Tomoyuki Koshida, Takahiro Arakawa, Hiroyuki Kudo, Hirokazu Saito, Kazuyoshi Yano, Kohji Mitsubayashi

**Affiliations:** 1 Department of Advanced Sciences and Technology for Biomedical Sensors, Graduate School of Medical and Dental Sciences, Tokyo Medical and Dental University, 1-5-45 Yushima, Bunkyo-ku, Tokyo 113-8549, Japan; E-Mail: miya.bdi@tmd.ac.jp; 2 Department of Biomedical Devices and Instrumentation, Institute of Biomaterials and Bioengineering, Tokyo Medical and Dental University, 2-3-10 Kanda-Surugadai, Chiyoda-ku, Tokyo 101-0062, Japan; E-Mails: arakawa.bdi@tmd.ac.jp (T.A.); kudo.bdi@tmd.ac.jp (H.K.); 3 Graduate School of Bionics, Tokyo University of Technology, 1404-1, Katakura-machi, Hachioji-shi, Tokyo 192-0982, Japan; E-Mails: t.koshida@hotmail.co.jp (T.K.); yano@bs.teu.ac.jp (K.Y.); 4 Department of Mechanical Engineering, Tokyo National Collage of Technology, 1220-2 Kunugida-machi, Hachioji-shi, Tokyo 193-0997, Japan; E-Mail: h.saito@tokyo-ct.ac.jp

**Keywords:** *E. coli* O157:H7, fluorescence, immunoassay, optical fiber, flow cell

## Abstract

Dynamic fluoroimmunoassay with a flow-through system using optical fiber probes consisting of polystyrene was developed and applied to a quantitative detection of *E. coli* O157:H7. The system measures *E. coli* as fluorescence of sandwich-type immune complexes formed by capture antibodies immobilized on the surface of the probe, *E. coli* cells, and fluorescently labeled detection antibodies. Excitation was carried out using an evanescent wave from the probe. Resulting fluorescence recoupled into the probe was detected by a photodiode. The assay system was constructed with a flow cell which was available for sequential injection of experimental reagents. *In vitro* characterization was performed using the flow cell, and the calibration range of *E. coli* O157:H7 was from 10^3^ to 10^7^ cells/mL. The measurement for each sample was completed within 12 min. Furthermore, it was also possible to estimate the concentrations of *E. coli* O157:H7 by the increasing rate of fluorescence during binding reaction of detection antibodies to antigens. This minimized the time for measurement down to 6 min. The system is suitable for rapid and direct determination for microorganisms or bacteria in food, clinical, and environmental sources.

## 1. Introduction

*Escherichia coli* (*E. coli*) is one of the normal flora of humans and animals that is known as an opportunistic pathogen. Although most *E. coli* are not harmful, some strains (e.g., O157:H7) cause serious clinical symptoms. It has been reported that human infection caused by *E. coli* O157:H7 occurs from a low-dose ingestion of the bacteria, even less than 100 organisms [1]. *E. coli* O157:H7 produce a toxin related to Shiga toxin of *Shigella dysenteriae* [2] and it causes severe life-threatening illness such as hemorrhagic colitis or hemolytic uremic syndrome [3,4]. Major sources of *E. coli* O157:H7 infections are contact with infected animals, unchlorinated water, unpasteurized milk, ground beef, fruit and vegetables [5]. Many developed countries including United States, Canada, Europe, Australia, and Japan have suffered from massive food-poisoning outbreaks caused by *E. coli* O157:H7 [6,7].

Traditionally, detection of *E. coli* O157:H7 is carried out by so-called serotyping which tests for specific antigens on the surface of the bacteria [8] after culturing of the stool on selective agar media for a long duration [9]. Although it is a highly sensitive and specific method, it is time consuming and less quantitative. These methods also require a special facility or laboratory that is equipped with the appropriate apparatus and expertise [10,11]. It usually takes at least one week for completing the above process. Therefore, an on-site method for rapid *E. coli* O157:H7 determination is strongly required. In addition, high sensitivity and high selectivity are needed for treatment and prevention of outbreaks.

Recently, several techniques for highly sensitive *E. coli* O157:H7 detection using fluorescence labels [12,13], immune-magnetic beads [14,15,16], DNA and RNA probes [17,18], quartz crystal microbalance techniques [19], surface plasmon resonance [20,21], polymerase chain reaction-based analysis [22,23] and flow cytometry [24] have been developed. In terms of user friendliness and field portability, sensors that rely on enzyme-linked immunosorbent assay or fluorescence immunoassay are proposed as a promising candidate of on-site *E. coli* O157:H7 detection.

A fiber sensing platform based on an evanescent field is of great interest in the field of biochemical and biological measurement [25,26,27,28,29,30]. This sort of sensor has been applied in the detection of a wide variety of pathogens such as staphylococcal enterotoxin B and *E. coli* O157:H7, *etc.* [31]. They are useful for direct and real-time detection of bacteria in foods by simply inserting the fiber-optic sensor. Here, sandwich immunoassay that uses tapered fiber-optic probes coated with capture antibodies and fluorescent dye-labeled antibodies was employed. *E. coli* O157:H7 concentrations could be determined by measuring the dye which was labeled by immune complexes in “sandwich” form. The concentrations also could be predicted by the rate of fluorescence increase during immunoreactions between *E. coli* O157:H7 and antibodies labeled with fluorescent dye.

In this paper, we introduce a new design of the fiber-optic fluorescent immunoassay system for rapid detection of *E. coli* O157:H7 using a flow cell that enables “in-reaction” measurement. In previous works, *E. coli* O157:H7 was detected using the fiber-optic fluorometric assay system which was one of novel immunological measurement techniques using the evanescent wave [32,33,34]. However, it was still time consuming because of the rate of transfer of the probe from the reaction unit to the measurement unit before and after the reaction during the measurement. Therefore, we employed the flow cell which has liquid inlets and an outlet for immunological reagent to carry out flow-through measurement of *E. coli* O157:H7. The design of a flow cell allows “in-reaction” measurement that is a measure of fluorescence during the binding reaction of detection antibodies to antigens. The flow-through system enabled rapid estimation of the antigen concentrations by analysis of the increasing rate of fluorescence. In addition, the minimized and automated system was suitable for household use. The flow-through system will therefore be useful for a bacteria test in daily life.

## 2. Experimental

### 2.1. Reagents

*E. coli* O157:H7 (#50-95-90, Positive Control, 3.76 × 10^9^ cells/mL) and anti-*E. coli* O157:H7 antibody (#01-95-90, ATCC43894, Goat-Poly) were purchased from Kirkegaard and Perry Laboratories Inc. *E. coli* O157:H7 standards (10^1^ to 10^7^ cells/mL) and other bacterial solutions were prepared by diluting a concentrated solution with the dilution buffer (phosphate buffer with 0.1% Triton X-100). Bovine serum albumin (BSA) was purchased from Itoham foods Inc. Cyanine 5 (Cy5) which is one of fluorescent dyes was labeled to anti- *E. coli* antibodies using an antibody labeling kit (#PA35000, Cy5-Ab labeling kit, Amersham Biosciences UK Ltd.). Antibodies are labeled according to the following method. One milliliter of antibody, coupling buffer and reactive dye solution are mixed and incubated at room temperature for 30 min with additional mixing approximately every 10 min. When conjugation of dye to antibody ends, the antibody-labeled mixture is divided by using a gel filtration column. Cy5-labeled antibody is obtained by removing the free dye. All other chemicals were of reagent grade.

### 2.2. Construction of Flow-Through System for *E. coli* O157:H7 Detection

**Figure 1:**
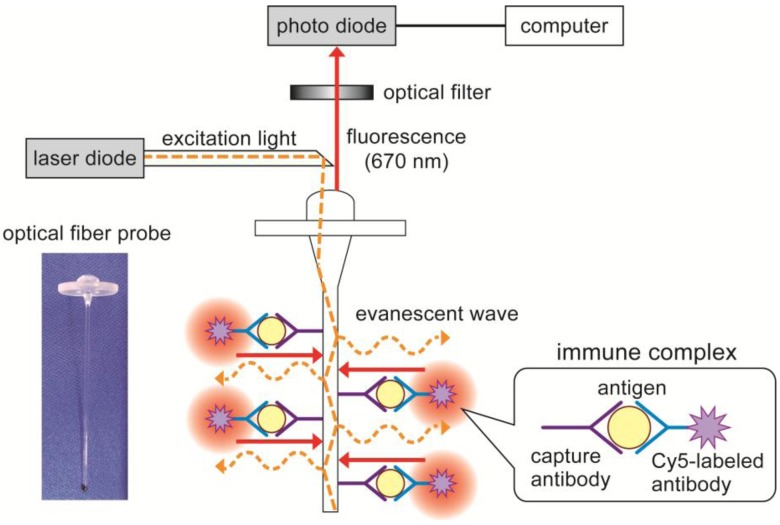
Principle of the fluoroimmunoassay system using a fiber-optic probe. A fluorescence-labeled antigen–antibody complex was made on the fiber surface. The excitation light (650 nm laser light) was coupled to the optical fiber probe, then fluorescence dye was excited by the evanescent wave that arose on the fiber. The fluorescent light (670 nm) was detected by a photodiode.

**Figure 2:**
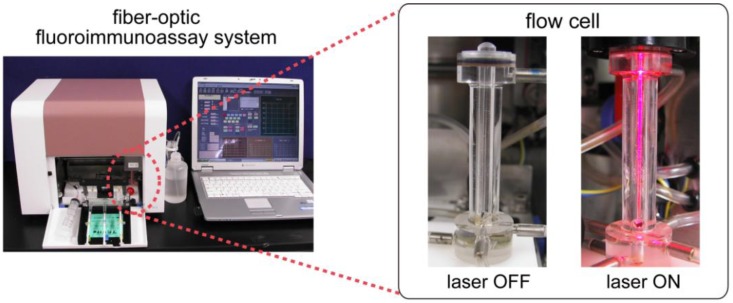
Fiber-optic fluoroimmunoassay system consisting of a flow-through cell. The flow cell is made from poly(methyl methacrylate) and the capacity is 350 μL.

The fluorometric assay system (PU6102) and polystyrene optical fiber probes (4 cm in length and 0.78 mm in diameter) were contributed by Canon Chemicals Inc. One end of the probe had a lens to effectively collect the excitation light and to guide it to the probe. Figure 1 shows the principle of the fluoroimmunoassay system for *E. coli* O157:H7. The measurement was performed using the customized PU6102 fluorometric assay system with the flow cell. The assay principle is based on a sandwich immunoassay, using optical fiber probes coated with capture antibodies and Cy5-labeled antibodies for fluorometric detection. The excitation light (λ = 650 nm) from a laser diode (LD) is coupled to the optical fiber probe, and the light is transmitted in the probe by total internal reflection. Then the Cy5 florescent molecules near the probe surface are excited by an evanescent light at the surface of the probe. *E. coli* O157:H7 is measured as fluorescence of Cy5 using a photodiode. Figure 2 shows the appearance of the customized PU6102 system and specially designed flow cell. The flow cell is made from poly(methyl methacrylate) and the capacity is 350 μL (except paths of flow).

**Figure 3:**
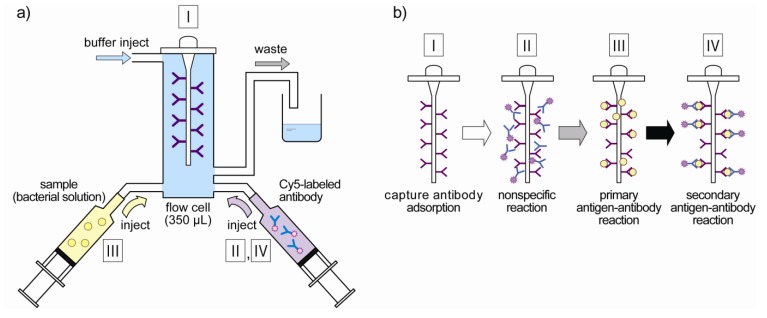
(**a**) Design of flow-through fluoroimmunoassay system. Bacteria solution and fluorescently labeled antibody are injected with syringes to the flow cell according to each measurement step. (**b**) The measurement step of the fiber-optic fluoroimmunoassay system. Fluorescent intensities are measured every measurement step, and the concentrations of *E. coli* O157:H7 are quantified.

Figure 3(a) represents the composition of the flow-through measurement system. *E. coli* O157:H7 cells and Cy5-labeled antibodies are put in the syringe by 800 µL and the optical fiber probe adsorbed with the capture antibodies is set in the flow cell. By controlling the injecting or collecting reagents in syringes, the antigen–antibody reaction is done and immune complexes are formed on the probe surface. Preliminarily, capture antibodies were immobilized by dipping the probe in the 100 μL of capture antibody solution, which contained 1.0 mg/mL capture antibodies, at 4 °C for 15 h. Then the probe was rinsed with phosphate buffer (PB; pH 7.4) and incubated with 1% BSA-PB to block the nonspecific binding site.

In this study, fluorescent intensities were measured at every step as shown in Figure 3(b) to detect the fluorescence which is specific to *E. coli* O157:H7. Fluorescence of capture antibodies adsorbed to the optical fiber probe was measured as baseline levels (STEP I). Then, the probe was incubated with Cy5-labeled antibodies for 5 min and fluorescence was read to define the background signal attributable to nonspecific binding of antibodies (STEP II). The probe was incubated with *E. coli* O157:H7 standards for 5 min to allow capture antibodies on the probe surface to bind with the cell-surface antigens (primary antigen–antibody reaction), and fluorescence was detected to measure any changes in background fluorescence (STEP III). Finally, the probe was incubated with Cy5-labeled antibodies again for 5 min to form sandwich-type immune complexes on the surface of the probe (secondary antigen–antibody reaction), and fluorescence specific to *E. coli* O157:H7 was measured during and after binding reaction of Cy5-labeled antibodies (STEP IV). Concentrations of *E. coli* O157:H7 were quantified in two different methods. One was the fluorescence at the end of step IV (*i.e*., after whole reactions in the measurement), and the other was the “in-reaction” measurement by analyzing the rate of fluorescence increase at step IV. The limit of detection was defined as three times the standard deviation of the blank signal.

### 2.3. Characterization of the Flow-Through System

*E. coli* O157:H7 standards were tested using the flow-through system in the range of 10^1^ to 10^7^ cells/mL (in PB with 0.1% Triton X-100), and the calibration range of the system for *E. coli* O157:H7 was determined. The variability of output signal by the individual difference of the optical fiber probes was determined by measuring fluorescent intensities using five different probes in each concentration. In addition, to determine the influence on the measurement by other bacteria, antigens measured were not only *E. coli* O157:H7 but also other bacteria or a mixture of *E. coli* O157:H7 and another. Bacteria used for the investigation were *Staphylococcus* sp., *Vibrio* sp. and nonpathogenic *E. coli* IAM12119 (all provided by H. Endo, Tokyo University of Marine Science and Technology).

## 3. Results and Discussion

### 3.1. Measurement of *E. coli* O157:H7

**Figure 4:**
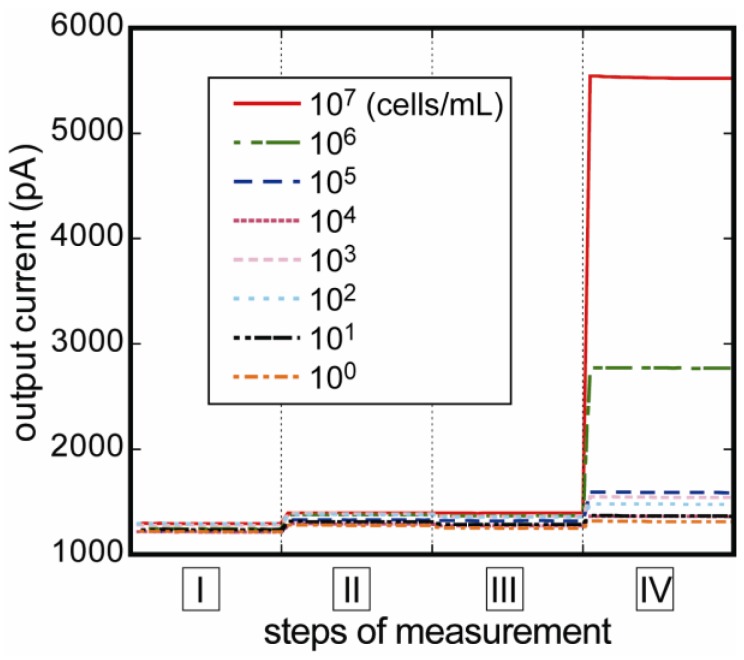
Changes in fluorescent intensities of response to *E. coli* O157:H7 concentrations. The signal of nonspecific reaction was negligibly low at step II, and stable current values at every four measurement steps were obtained immediately.

**Figure 5:**
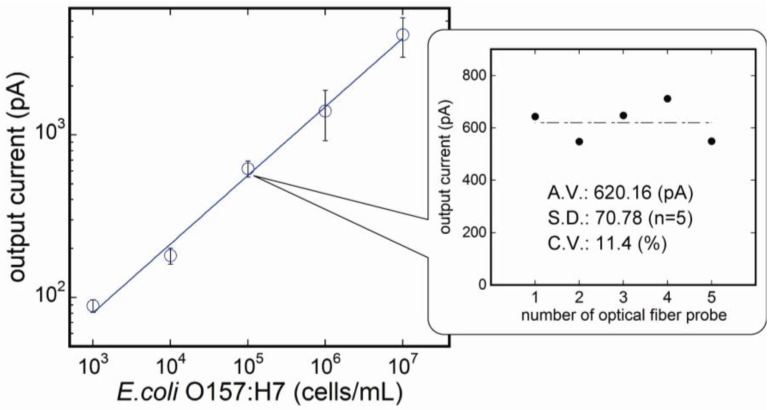
Calibration curve for *E. coli* O157:H7 measured by the fluoroimmunoassay system (n = 3). *E. coli* O157:H7 concentration of 10^7^ cells/mL gave the strongest signal enhancement, and the signal strength decreased as the antigen concentration decreased.

Figure 4 shows the variation of fluorescent intensity corresponding to *E. coli* O157:H7 concentrations at each measurement step. No significant nonspecific reaction was confirmed at step II, and the current was sufficiently stable throughout the measurement. As the *E. coli* O157:H7 concentration increased, the fluorescence signal also increased. Calibration curve measured by the flow-through measurement system is shown in Figure 5. The calibration curve was made from data which had fluorescent difference between steps III and IV. As a result, the highest fluorescence was obtained at 10^7^ cells/mL, and the lower detection limit was determined to be 10^3^ cells/mL. The coefficient of determination (R2) was 0.999 deduced by regression analysis as shown by the following equation:

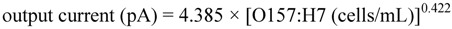
(1)

The reaction time in the Cy5-labeled antibodies and *E. coli* O157:H7 standards was 5 min, respectively, and the measurement time of each step was 20 s. Therefore, the time required to complete assay (without adsorption of capture antibodies to probes) was about 16 min and 20 s. The immunoassay system was less time consuming to measure bacteria than a conventional method, which needed growth and culturing bacteria and long reaction time for each immunological reagent (such as samples, detection antibodies and substrate solution). The system shows almost the same sensitivity as conventional methods [35] and does not require any sample preparation. In addition, the system has the properties of being simple to use and bearing a compact design, therefore our system is well suited for on-site testing for *E. coli* O157:H7.

### 3.2. Reproducibility of the Fluoroimmunosensor

To evaluate the variability of the signals by the individual difference of the optical fiber probes, *E. coli* O157:H7 solutions of the same concentration (10^5^ cells/mL) were measured using five different optical fiber probes. Results are shown in the inset to Figure 5. The system showed sufficient reproducibility, while there is a little variability among probes (c.v. 11.4 %). Probes do not require any complicated treatments to absorb capture antibodies to the probe surface, and, therefore, the results show not only reproducibility, but also simplicity of the measurement technique. To improve reproducibility of the system, it is considered that chemical modification of the surface of the probe is beneficial for uniform binding of capture antibodies, and eventually reproducibility of measurement of *E. coli* O157:H7.

### 3.3. Fluorescence Monitoring and Measuring of *E. coli* O157:H7 by the Flow-Through System

**Figure 6:**
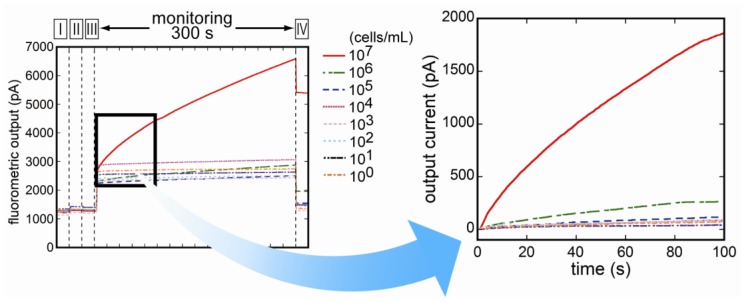
Monitoring of fluorescence during the entire time of measurement. The amount of fluorescent intensity of each concentration of *E. coli* O157:H7 increased during binding reaction of Cy5-labeled antibodies to cell-surface antigen.

**Figure 7:**
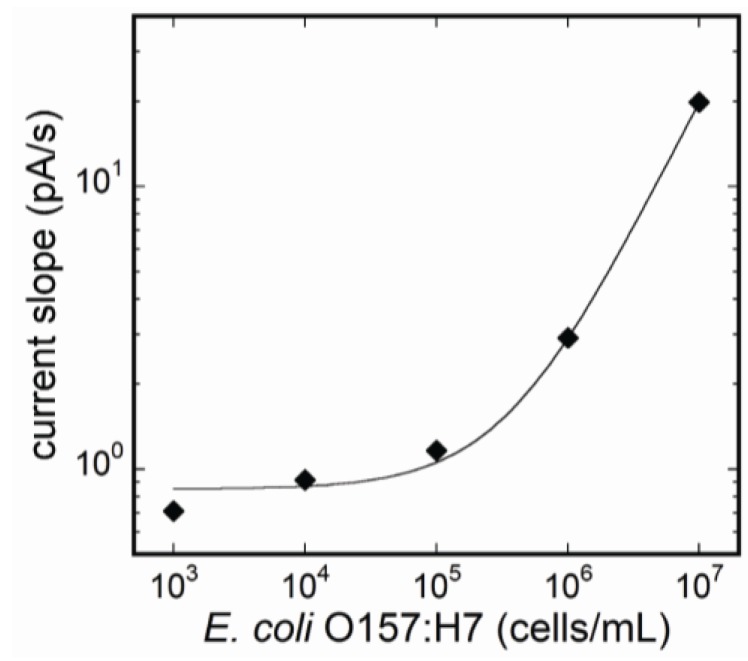
Calibration curve by calculating the rate of fluorescence increase during binding reaction of Cy5-labeled detection antibodies. The concentrations of *E. coli* O157:H7 were quantified without reducing sensitivity of the fluoroimmunoassay system.

The flow-through system was constructed by employing the flow cell linked to the optical detection unit. Supplying the reagents sequentially into the flow cell, fluorescence was able to be measured throughout the immunoreaction. Changes of fluorescence during binding reaction of Cy5-labeled antibodies to *E. coli* O157:H7 cells were monitored. Figure 6 represents the changes of the fluorescence for the entire time of measurement. During binding reaction of Cy5-labeled antibodies, the amount of fluorescent intensity of each concentration of *E. coli* O157:H7 increased as the reaction proceeded. In addition, the increasing rate of the fluorescence was associated with the concentration of *E. coli* O157:H7. Figure 7 shows the relation between antigen concentrations, and each plot in the figure represents the increased rate of fluorescence at 1 min after injection of Cy5-labeled antibodies. The calibration range was almost the same as the quantification method described above. The coefficient of determination (R) was 1.000 by the regression analysis as shown by the following equation:

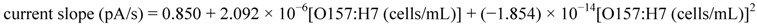
(2)

By monitoring fluorescent changes in incubating reaction at step IV, the time of the assay was completed within 6 min, excepting the confirmation of nonspecific reaction at step II. The assay was dramatically shortened from that of the conventional detection method for *E. coli* O157:H7 detection. 

Furthermore, the immunoassay is able to measure samples every 6 minutes by replacing the optical fiber probe with a new one under flow condition. Therefore, the sensor was available for semi-real-time determination of pathogens in food or measurement one sample after another. As discussed above, *E. coli* O157:H7 is highly infectious and it only needs a few bacteria to cause illness. The result suggests that, due to its simplicity, sensitivity and swiftness, the fiber-optic fluoroimmunoassay system is suitable for detection of *E. coli* O157:H7 on-site to prevent illness caused by food-borne or water-borne pathogens. 

### 3.4. Selectivity of the Fluoroimmunosensor for *E. coli* O157:H7

**Figure 8:**
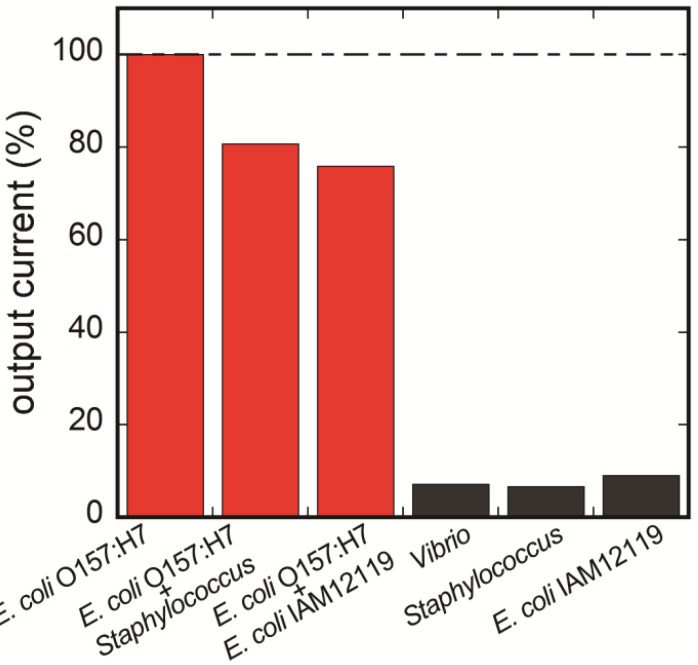
Selectivity of the fiber-optic fluoroimmunoassay to other food-borne pathogens. Positive signals were observed with only antigens containing *E. coli* O157:H7.

For testing selectivity to other food-borne pathogens, six different bacteria solutions including two-in-one mixture solutions were prepared. The antigen solutions were prepared using nonpathogenic *E. coli* IAM12119 (10^5^ cells/mL), *Staphylococcus* sp. (10^5^ cells/mL), *Vibrio* sp. (10^5^ cells/mL) and *E. coli* O157:H7 (10^5^ cells/mL). In addition, two types of mixtures of bacteria were prepared; the one was compounded from *E. coli* O157:H7 (10^5^ cells/mL) and *Staphylococcus* sp. (10^5^ cells/mL), and the other was compounded from *E. coli* O157:H7 (10^5^ cells/mL) and nonpathogenic *E. coli* IAM12119 (10^5^ cells/mL). Fluorescent intensities were measured as described above in each of antigen solutions. Signals of each antigen obtained from differing values between steps III and IV are shown in Figure 8. For comparison, output current of *E. coli* O157:H7 sample was defined as 100%. 

Antigens that contain no *E. coli* O157:H7 provided little positive signal in comparison to the negative control, whereas antigens containing *E. coli* O157:H7 provided almost the same signal values. This result showed that the immunoassay was less affected by the presence of any other food-borne bacteria or even nonpathogenic *E. coli*. The reason for slightly dropped signals of mixture samples included that the capture antibody had little cross-reactivity with other bacteria. On the other hand, we had already measured milk samples which were artificially contaminated with the cell of *E. coli* O157:H7 [34]. In the future, this immunoassay system will be aimed to use for food-borne or water-borne bacterial samples in the aqueous matrix. To this end, additional research to improve the sensitivity and selectivity is needed. For instance, the high-density immobilization of capture antibodies to the probe or the use of low cross-reactive antibodies should lead the measurement to simple and rapid detection of bacteria without cell isolation or culturing.

## 4. Conclusions

A fiber-optic immunoassay system with flow-through system for *E. coli* O157:H7 was constructed and tested. The measurement time was reduced to 12 min by employing the flow cell. Nevertheless, the sensitivity was not decreased. The calibration range for *E. coli* O157:H7 was from 10^3^ to 10^7^ cells/mL, which was similar to the previous system. The system also showed sufficient reproducibility regardless of individual differences in the optical fiber probes. We also monitored fluorescence changes during binding reaction of Cy5-labeled detection antibodies to *E. coli* O157:H7. The concentrations of pathogens were quantified by calculating the fluorescence increase, and as a result, the total assay time was shortened. The selectivity of the system was tested by using nonpathogenic *E. coli* O157, *Staphylococcus* sp. and *Vibrio* sp. Positive signals of fluorescence were observed with only antigens containing *E. coli* O157:H7. Therefore, the results presented in this paper prove that the immunoassay is simple, reliable, specific and rapid. In the future, we are going to improve the system in order to get a higher output signal for the on-site use by increasing the adaption efficiency of capture antibodies to the probe. In addition, the sensor system may allow for adapting to the detection of other food- and water-borne pathogens by choosing the appropriate capture antibody for the pathogen.
